# Trends in Epidemiology of Esophageal Cancer in the US, 1975-2018

**DOI:** 10.1001/jamanetworkopen.2023.29497

**Published:** 2023-08-22

**Authors:** Gladys M. Rodriguez, Dylan DePuy, Mayada Aljehani, Jeffrey Bien, Jerry S. H. Lee, David H. Wang, Albert Y. Lin

**Affiliations:** 1 Divisions of Hematology and Medical Oncology, Stanford University School of Medicine, Stanford, California; 2 Lawrence J. Ellison Institute for Transformative Medicine, Los Angeles, California; 3 Permanente Medical Group, Santa Clara, California; 4 Departments of Medicine, Chemical, and Material Sciences and Quantitative and Computational Biology, University of Southern California, Los Angeles; 5 Esophageal Diseases Center, Hamon Center for Therapeutic Oncology Research, Division of Hematology and Oncology, Simmons Cancer Center, UT Southwestern Medical Center, Dallas, Texas; 6 VA North Texas Health Care System, Dallas; 7 VA Palo Alto Health Care System, Palo Alto, California; 8 Stanford University School of Medicine, Stanford, California

## Abstract

**Importance:**

Esophageal cancer (EC) is the 7th most common cancer worldwide and 14th in the US. More data are needed to study the changing incidence patterns of its 2 primary histologic subtypes, squamous cell carcinoma of the esophagus (SCE) and adenocarcinoma of the esophagus (ACE).

**Objective:**

To examine temporal trends in incidence rates of EC, ACE, and SCE from 1975 through 2018.

**Design, Setting, and Participants:**

In this population-based cross-sectional study, data were derived from 9 Surveillance, Epidemiology, and End Results (SEER) registries from January 1975 through December 2018 and from all 21 registries for January 2000 through December 2018 for patients with a diagnosis of EC from 1975 through 2018 (*International Classification of Disease—Oncology, Third Edition *codes). Age-adjusted incidence rates (AAIRs) of EC, ACE, and SCE were calculated. The timing and magnitude of the annual percentage change (APC) in incidence were examined using Joinpoint regression analyses. Data analysis was started in 2021 and updated and completed in 2023.

**Main Outcome and Measures:**

The APC for age-adjusted EC incidence rates as stratified by histology, anatomical location, stage, sex, age, race and ethnicity, and geographic region.

**Results:**

A total of 47 648 patients with a diagnosis of EC were retained for analysis. These included 22 419 (47.1%) with a diagnosis of SCE, 22 217 (46.6%) with ACE, and 3012 (6.3%) with other subtypes. The AAIR for EC changed from 4.14 per 100 000 population in 1975 to 4.18 in 2018, AAIRs of SCE declined from 3.06 in 1975 to 1.15 in 2018 as well as for ACE, and AAIRs increased from 0.42 in 1975 to 2.78 in 2018. From 1975 through 2004, EC incidence significantly increased (APC, 0.53; 95% CI, 0.4 to 0.7) but significantly decreased (APC, −1.03; 95% CI, −1.3 to −0.7) from then until 2018. The APC of SCE significantly continued to decline (−2.80, 95% CI, −3.0 to −2.6), and ACE increased from 2000 to 2006 (APC, 2.51; 95% CI, 1.0 to 4.0) but has since stabilized from 2006 to 2018.

**Conclusions and Relevance:**

The results of this cross-sectional study suggest that the incidence of EC modestly declined since 2004 and that the incidence of SCE continued to decline while the incidence rate of ACE plateaued for more than a decade. Understanding factors associated with plateaued rates of ACE may help inform public health interventions.

## Key Points

**Question:**

How did the incidence patterns of esophageal cancer (EC) and its 2 primary histologic subtypes, squamous cell carcinoma and adenocarcinoma (ACE), change from 1975 to 2018?

**Findings:**

In this population-based cross-sectional study of 47 648 patients with EC, the overall annual percentage change (APC) in incidence of EC increased significantly from 1975 to 2004 by 0.53 and then modestly declined from 2004 to 2018 with an APC of −1.25. From 2000 to 2018, squamous cell carcinoma incidence significantly declined, with an APC of −2.80, while ACE incidence increased from 2000 to 2006 with an APC of 2.51 before stabilizing from 2006 to 2018.

**Meaning:**

The results of this cross-sectional study suggest that understanding factors associated with plateaued rates of ACE may help inform public health interventions.

## Teaser

This cross-sectional study examines temporal trends in incidence rates of esophageal cancer, adenocarcinoma of the esophagus, and squamous cell carcinoma of the esophagus from 1975 through 2018.

## Introduction

Esophageal cancer (EC) is the seventh most common cancer and the sixth leading cause of cancer mortality worldwide, with about 544 000 deaths reported in 2020.^1^ The 5-year survival for all stages of EC combined is around 20%.^2^ In the US, EC is the 14th most common cancer, with an estimated 21 560 new diagnoses and 16 120 expected deaths in 2023.^2^ Men are at higher risk of developing EC than women.^2^ Management of EC has evolved to include immunotherapy as part of the standard treatment for early and advanced stages.^3^ Squamous cell carcinoma of the esophagus (SCE) and adenocarcinoma of the esophagus (ACE) are the 2 most common histologic subtypes of EC. Risk factors for EC include gastroesophageal reflux disease (GERD), Barrett esophagus, obesity, metabolic syndrome, alcohol use, and tobacco smoking.^4^ Studies have shown that obesity is likely to be associated with ACE through an independent and a GERD-dependent and Barrett esophagus–dependent mechanism.^5^

The incidence of SCE has steadily declined during the last few decades.^6^ In contrast, the incidence of ACE increased from the 1970s to 2006 from 3.6 cases per million to 25.6 cases per million, a 7-fold increase.^7^ A study spanning from 1997 to 2014 suggested that ACE incidence decreased or stabilized.^6^ Longer follow-up is needed to study the overall incidence patterns of EC, ACE, and SCE and provide a better delineation of the changes in trends in recent years. More data are also needed to investigate incidence patterns among subgroup populations in the US. The objective of this study was to conduct a retrospective population-based analysis to examine temporal trends in incidence rates of EC, ACE, and SCE from 1975 through 2018 using Surveillance, Epidemiology, and End Results (SEER) 9 (the registry with the longest follow-up duration) and SEER 21 (the registry that covers the largest US population registry) and provide an update on recent changes in incidence.

## Methods

### Data Source and Study Population

Data used in this study were derived from SEER of the National Cancer Institute, a population-based cancer database. For the period between 1975 through 2018, we used data from 9 SEER registries that covered 9.4% of the US population. From 2000 through 2018, data from all 21 SEER registries, which covered 36.7% of the US population, were used.^8^

Records for patients with a diagnosis of EC from 1975 through 2018 were retrieved from the SEER databases using the *International Classification of Disease—Oncology, Third Edition* (*ICD-O-3*).^9^ Cases with *ICD-O-3* morphology codes 8050 to 8082 were classified as SCE, those with codes 8140-8573 as ACE, and those with codes other than 8050 to 8082 and 8140-8573 as other subtypes. This study used deidentified SEER data; thus, institutional review board was not required and informed consent was waived.

### Study Covariates

Patient demographic characteristics included age group at diagnosis (<65 years, 65-75 years, and >75 years), race and ethnicity (Black, Hispanic, and non-Hispanic White), and sex. Race and ethnicity classification was based on electronic health records and is abstracted by SEER. Census regions were used to assign states for regional analysis.^10^ For analysis of stage variation, cases were classified by SEER stage (localized, regional, or distant) at diagnosis.^11^ Anatomical distribution was across 4 locations as defined by *ICD-O-3* topography codes: cervical esophagus (C15.0), upper thoracic portion (C15.3), middle thoracic portion (C15.4), and lower thoracic portion (C15.5).

### Statistical Analysis

**Figure 1.**
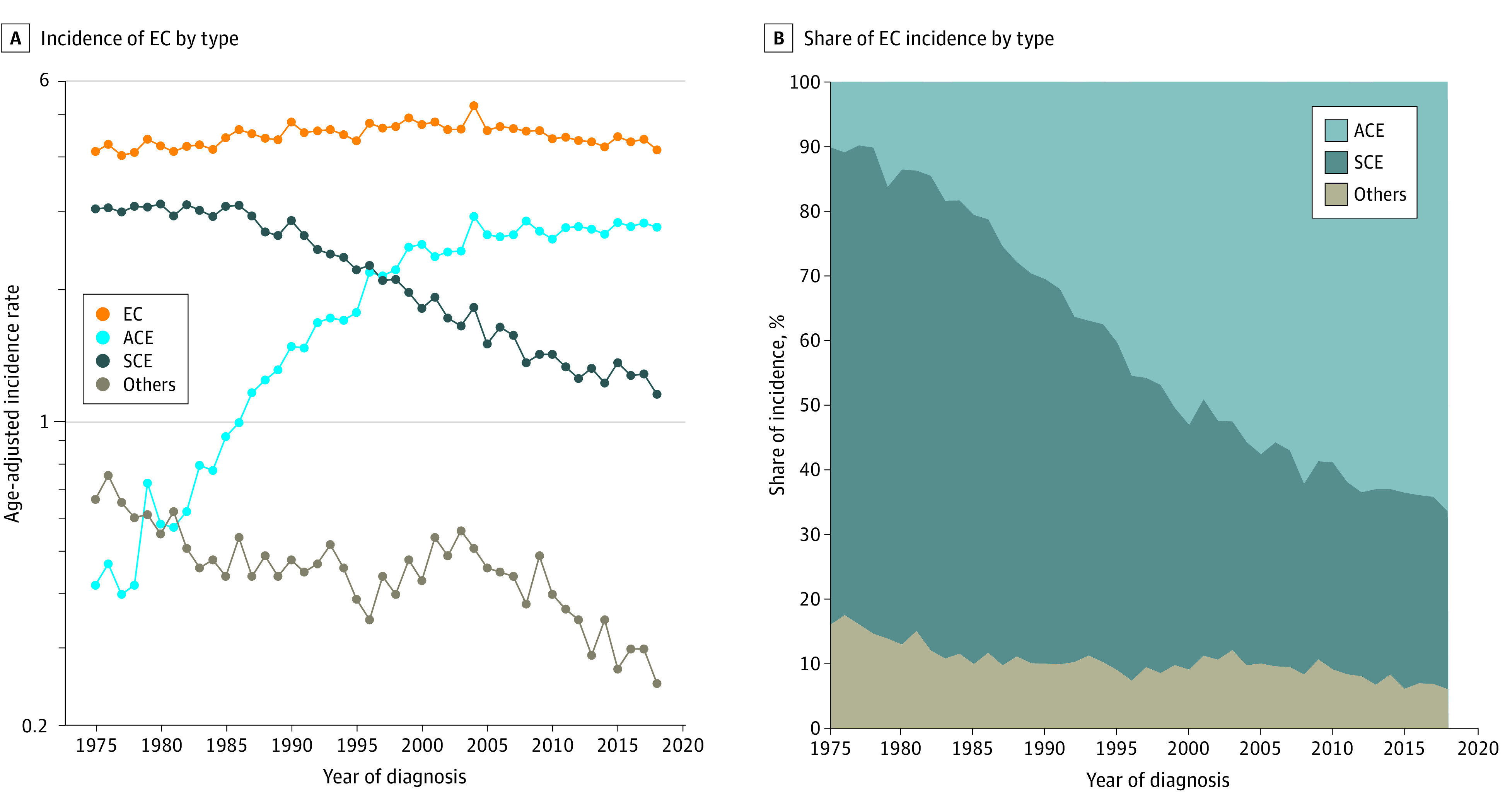
Incidence of and Percentage Share of Incidence of Esophageal Cancer (EC), Adenocarcinoma of Esophagus (ACE), Squamous Carcinoma of Esophagus (SCE), and Other Subtypes

Data analyses were conducted in a 2-step approach. In the initial step, age-adjusted incidence rates (AAIRs) for EC, ACE, and SCE were calculated using SEER proprietary statistical analysis software (SEER*Stat, version 8.3.9.2; National Cancer Institute). Incidence rates were computed per 100 000 individuals and age-adjusted to the 2000 US standard population. During the subsequent step, and using incidence rates obtained in the first step, the timing and magnitude of the annual percentage change (APC) in incidence trends over time were examined using the National Cancer Institute’s Joinpoint Trend Analysis Software, version 4.9.0.0. The software tests the statistical significance of a change in trend using a Monte Carlo permutation method (in this study, the overall significance level was set to .05 and number of permutations to 4499). A minimum and maximum of 0 and 3 joinpoints, respectively, were selected. Incidence was considered to be rising when the APC’s 95% CI was greater than 0 and falling when the APC’s 95% CI was less than 0. If the APC’s 95% CI included 0, the incidence trend was considered stable. All figures (except Figure 1B and the eFigure in Supplement 1, which do not depict rates) were prepared such that a 10° slope reflects a 1% annual rate of change.^12^ The reporting of study results followed the Strengthening the Reporting of Observational Studies in Epidemiology (STROBE) checklist for cross-sectional studies.

## Results

A total of 47 648 patients with a diagnosis of EC were retained for analysis. These included 22 419 (47.1%) with a diagnosis of SCE, 22 217 (46.6%) with ACE, and 3012 (6.3%) with other subtypes.

### Overall Incidence

The AAIR for EC overall changed modestly from 4.14 per 100 000 population in 1975 to 4.18 in 2018 despite large changes in the AAIRs of SCE (3.06 in 1975, 1.15 in 2018) and ACE (0.42 in 1975, 2.78 in 2018) (Figure 1A). As such, the histologic subtype responsible for most EC incidence has gradually shifted from SCE (73.91% of all EC incidence in 1975) to ACE (66.51% of all EC incidence in 2018) (Figure 1B).

**Figure 2.**
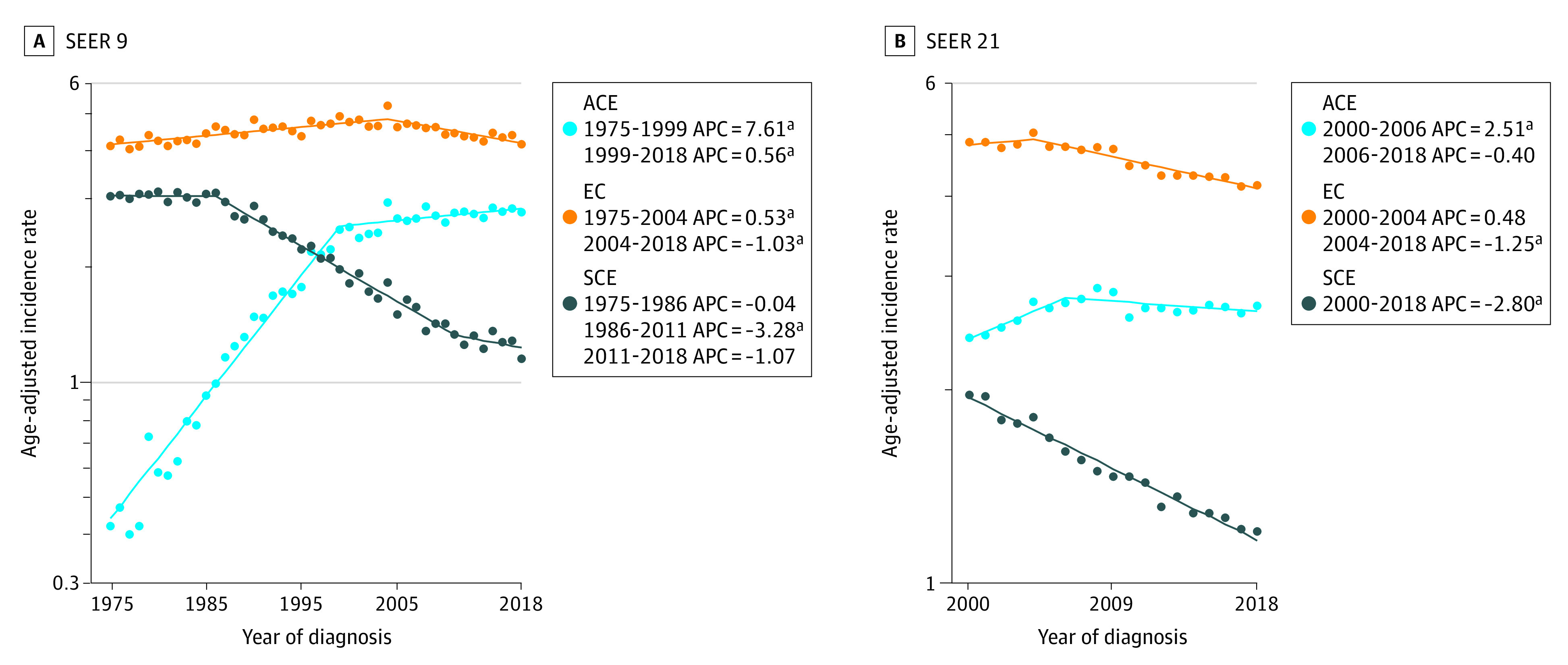
Joinpoint Analysis of Overall Esophageal Cancer (EC), Adenocarcinoma of Esophagus (ACE), and Squamous Carcinoma of Esophagus (SCE) Incidence APC indicates annual percentage change; SEER, Surveillance, Epidemiology, and End Results. ^a^*P *<.05.

Joinpoint models of EC, ACE, and SCE overall incidence from 1975 to 2018 (SEER 9) and 2000 to 2018 (SEER 21) are shown in Figure 2. SEER 9 suggests that EC incidence was increasing significantly from 1975 to 2004 (APC, 0.53; 95% CI, 0.4 to 0.7) and then declined significantly by 1.0% annually (95% CI, −1.3 to −0.7) until 2018. SEER 21 also points to EC incidence beginning to decline in 2004, although at a slightly faster rate (APC, −1.25; 95% CI, −1.5 to −1.0). In SEER 9, SCE incidence appeared stable from 1975 to 1986, declined from 1986 to 2011 (APC, −3.28; 95% CI, −3.5 to −3.0), and then stabilized after 2011. However, in SEER 21, SCE incidence continued to decline until 2018 (2000-2018: APC, −2.80; 95% CI, −3.0 to −2.6). A rapid rise in ACE incidence from 1975 to 1999 (APC, 7.61; 95% CI, 7.0 to 8.2) was observed in SEER 9, which slowed after 1999 (1999-2018: APC, 0.56; 95% CI, 0.1 to 1.0). Incidence of ACE stabilized in 2006 according to SEER 21 (2000-2006: APC, 2.51; 95% CI, 1.0 to 4.0) (Figure 2).

### Demographic Differences

Joinpoint models of EC, SCE, and ACE incidence from 2000 to 2018 (SEER 21) as stratified by age group, sex, and race and ethnicity are shown in the eFigure in Supplement 1. For EC overall, 4 of 48 groups exhibited increasing incidence for any period (non-Hispanic White male individuals overall, non-Hispanic White older than 75 years overall, male individuals older than 75 years overall, and non-Hispanic White male individuals older than 75 years). The fastest rate of increase was observed among both non-Hispanic White male individuals older than 75 years and non-Hispanic White individuals older than 75 years overall (APC, 3.3; 95% CI, 0.4 to 6.3) from 2000 to 2004. Among the older than 75 years group, 4 subgroups exhibited a joinpoint in incidence in 2004 that was followed by declining incidence: older than 75 years overall, non-Hispanic White overall, male individuals overall, and non-Hispanic White males overall. A similar trend was observed among non-Hispanic White male individuals younger than 65 years, non-Hispanic White male individuals overall, male individuals overall, and non-Hispanic White individuals overall (eFigure in Supplement 1).

For SCE, 42 of 48 groups saw declining incidence during the entire period. Groups that demonstrated statistically significant rapid rates of decline included all Hispanic male individuals (2015 to 2018: APC, −9.0 95% CI, −15.5 to −2.0), Hispanic male individuals aged 65 to 75 years (2010 to 2018: APC, −8.5; 95% CI, −11.9 to −4.9), and Black male individuals younger than 65 years (2000 to 2018: APC, −6.2; 95% CI, −6.9 to −5.5). However, the subgroup of Hispanic male individuals older than 75 years exhibited the fastest rates of decline, although it was not statistically significant (2000 to 2004: APC, −12.3; 95% CI, −24 to 1.2; 2016 to 2018: APC, −24.5; 95% CI, −51 to 16.5). This lack of significance may be attributed to the limited number of patients within that age group (eFigure in Supplement 1).

Incidence of ACE was stable among 32 of 48 groups during the entire period. The largest rate of increase was observed among non-Hispanic White individuals older than 75 years overall (APC, 7.2; 95% CI, 2.8 to 11.7) and non-Hispanic White male individuals older than 75 years (APC, 7.2; 95% CI, 2.7 to 11.9) from 2000 to 2004. The same 4 groups from the older than 75 years group that exhibited a shift in EC incidence overall in 2004 also did so for ACE incidence, after which incidence plateaued. Other groups exhibited similar trends, including all those of White male individuals, with segments beginning in 2000 and ending between 2004 and 2007 characterized by increases in incidence that were followed by segments until 2018 with relatively little change in or stabilized incidence (eFigure in Supplement 1).

### Stage Variation

**Figure 3.**
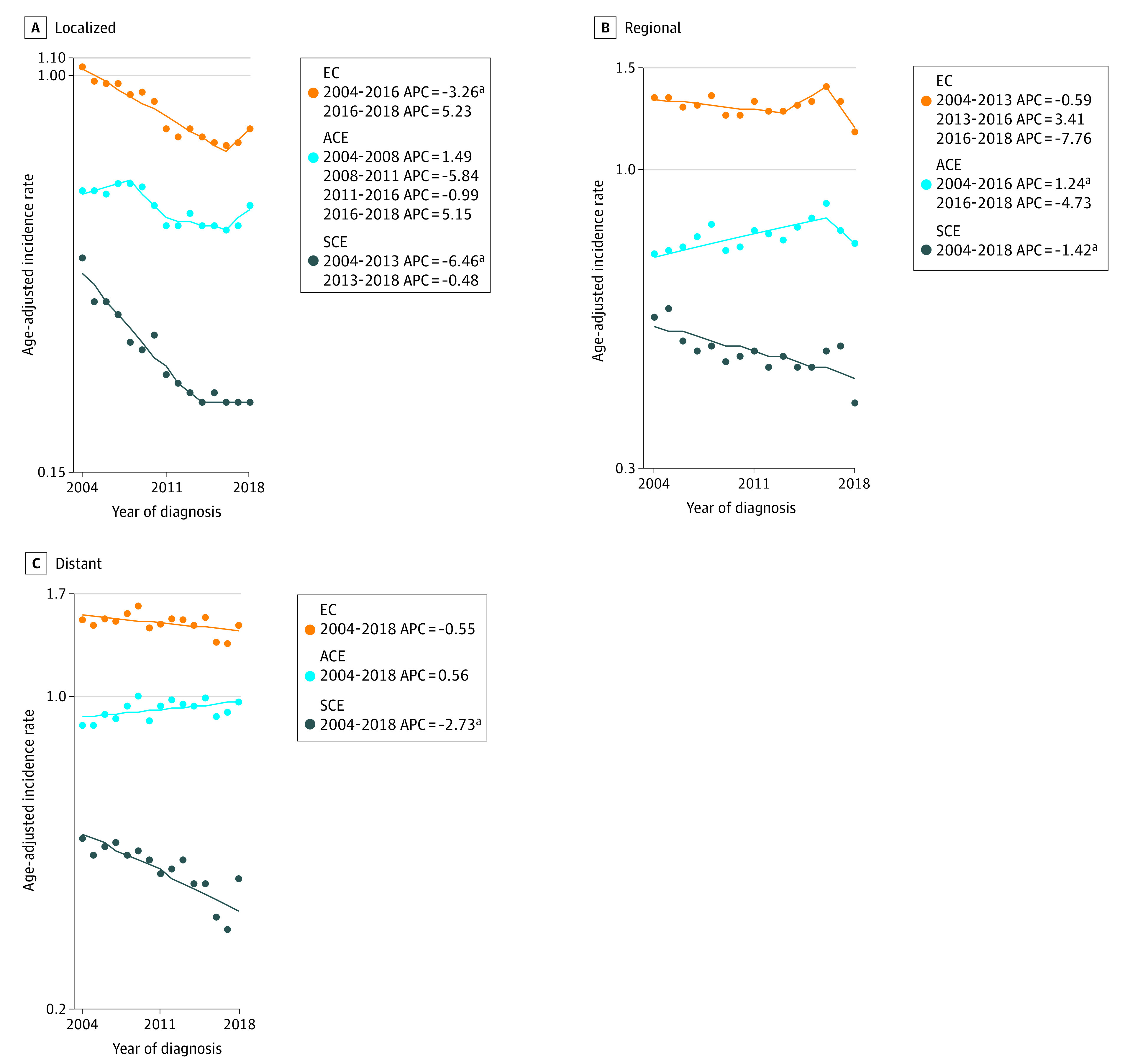
Joinpoint Analysis of Esophageal Cancer (EC), Adenocarcinoma of Esophagus (ACE), and Squamous Carcinoma of Esophagus (SCE) Incidence by Stage APC indicates annual percentage change. ^a^*P *<.05.

Data for EC, SCE, and ACE AAIRs by stage at diagnosis were available from 2004 to 2018 (SEER 21), and corresponding joinpoint models are shown in Figure 3. Incidence of localized EC was lower than regional and distant and declined from 2004 to 2016 by −3.26% per year (95% CI, −3.9 to −2.6). Incidence of ACE was stable. Localized SCE incidence declined from 2004 to 2013 at an APC of −6.46 (95% CI, −7.6 to −5.3) and then stabilized. Regional EC incidence was stable, SCE incidence fell by 1.42% annually (95% CI, −2.2 to −0.6) from 2004 to 2018, and ACE incidence rose from 2004 to 2016 (APC, 1.24; 95% CI, 0.5 to 1.9). Distant EC and ACE incidence were steady, although SCE incidence fell at an APC of −2.73 (95% CI, −3.7 to −1.7) from 2004 to 2018 (Figure 3).

### Anatomical Distribution

**Figure 4.**
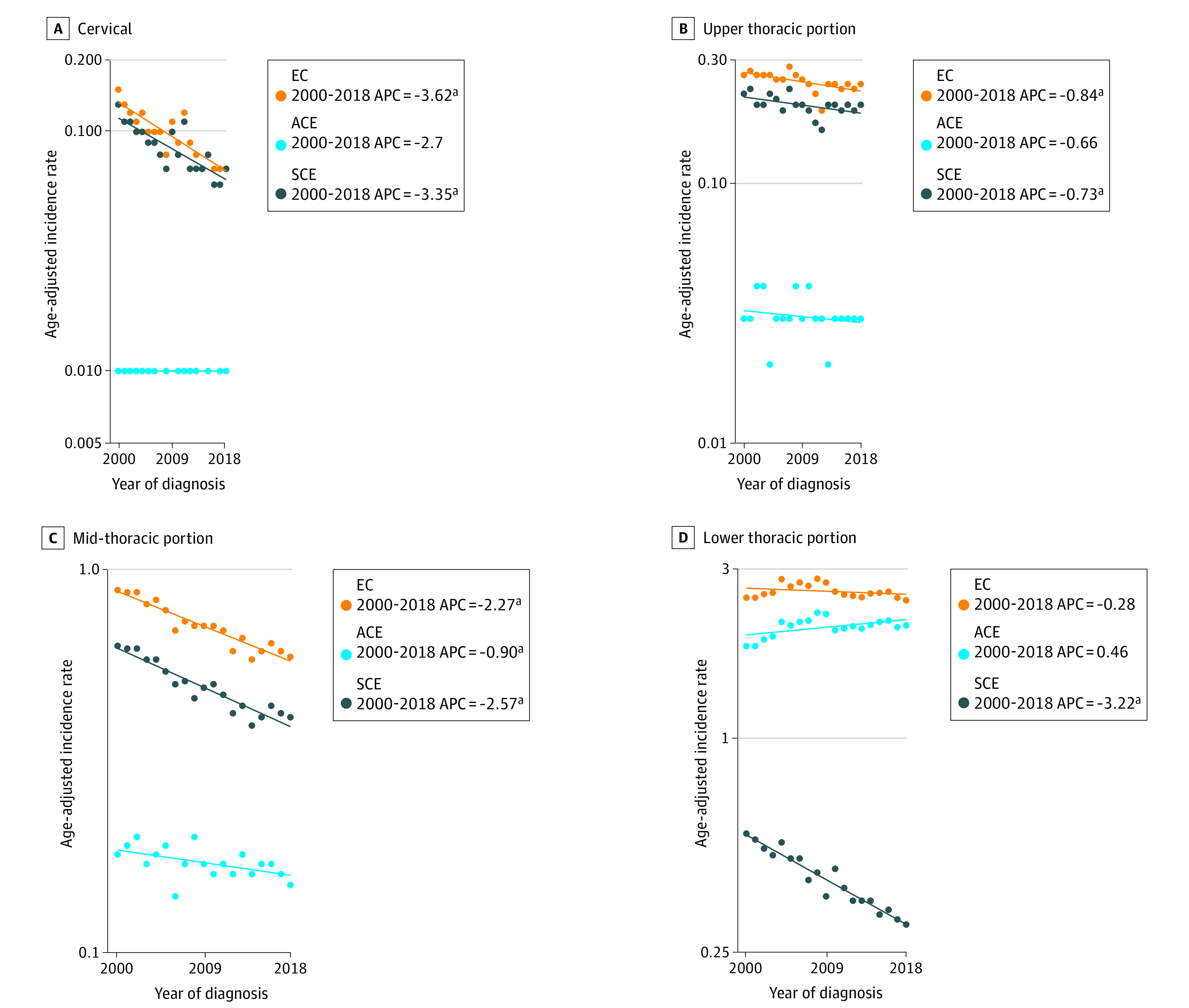
Esophageal Cancer (EC), Adenocarcinoma of Esophagus (ACE), and Squamous Carcinoma of Esophagus (SCE) Incidence by Anatomical Site of Origin APC indicates annual percentage change. ^a^*P *<.05.

Incidence trends of EC, ACE, and SCE by anatomical site of origin from 2000 to 2018 are shown in Figure 4. Incidence of EC originating in the cervical esophagus declined from 2000 to 2018 by 3.62% annually (95% CI, −4.61 to −2.63). This change was largely associated with the declining SCE incidence (2000 to 2018: APC, −3.35; 95% CI, −4.39 to −2.3), the predominant histology in the cervical esophagus. From 2000 to 2018, incidence of EC originating in the upper thoracic portion of the esophagus declined at an APC of −0.84% (95% CI, −1.42 to −0.25), SCE incidence declined at an APC of −0.73 (95% CI, −1.38 to −0.06), and ACE incidence was stable. Incidence of EC originating in the midthoracic portion of the esophagus declined by 2.27% annually from 2000 to 2018 (95% CI, −2.64 to −1.9). Midthoracic SCE incidence declined at an APC of 2.57 (95% CI, −3.01 to −2.13), and ACE incidence declined at an APC of −0.90 (95% CI, −1.57 to −0.22) from 2000 to 2018. Incidence of EC was highest for cases originating in the lower thoracic portion of the esophagus and was stable from 2000 to 2018. Lower thoracic ACE incidence was also stable, and SCE incidence declined by 3.22% annually (95% CI, −3.66 to −2.78) (Figure 4).

### Regional Incidence

**Figure 5.**
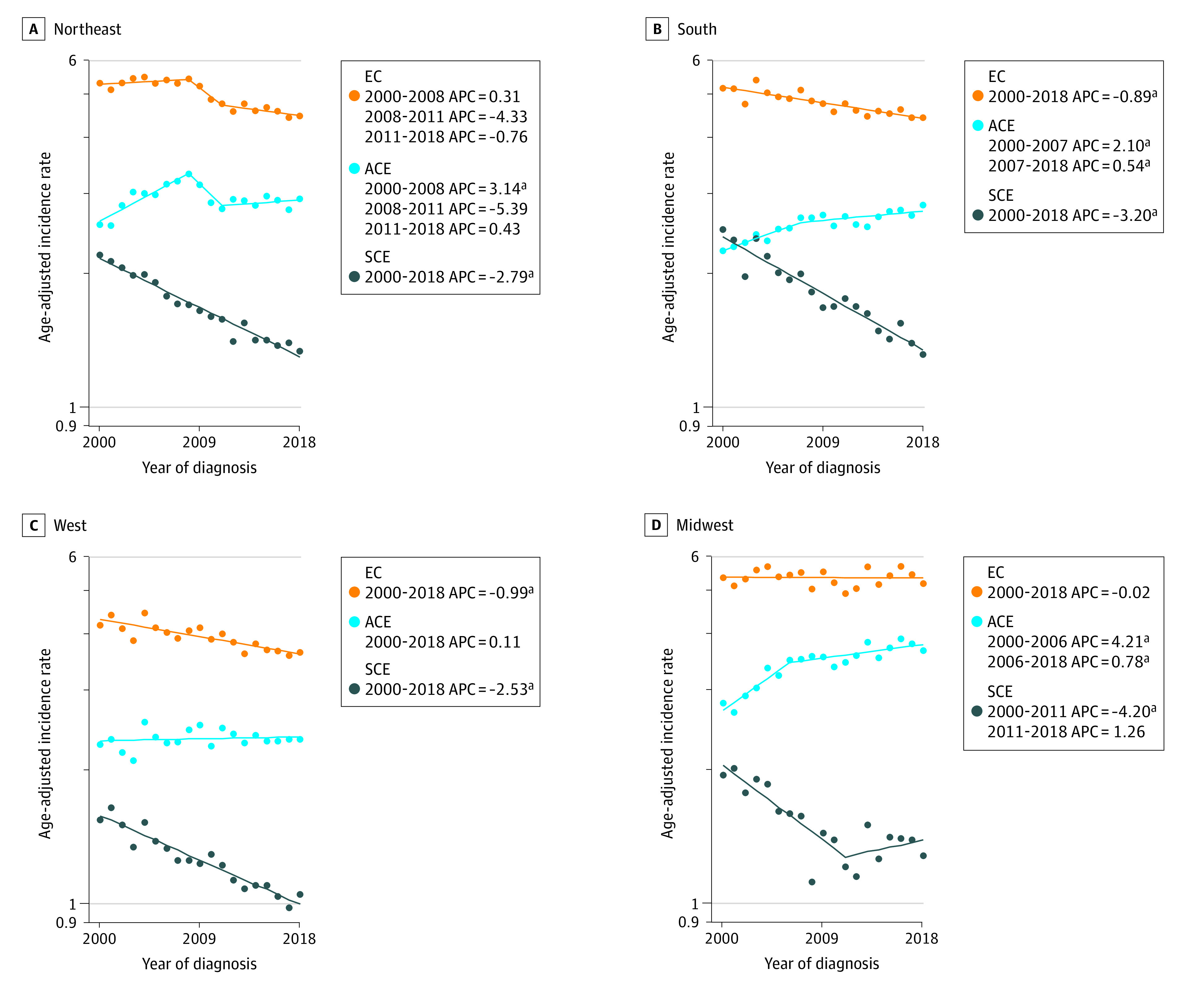
Joinpoint Analysis of Esophageal Cancer (EC), Adenocarcinoma of Esophagus (ACE), and Squamous Carcinoma of Esophagus (SCE) Incidence by Census Region APC indicates annual percentage change. ^a^*P *<.05.

Figure 5 depicts joinpoint models of EC, ACE, and SCE incidence patterns by region. Incidence was lowest in the West, where EC incidence declined by −0.99% annually (95% CI, −1.3 to −0.7), ACE incidence was stable, and SCE incidence declined at an APC of −2.53 (95% CI, −2.9 to −2.2) from 2000 to 2018. In the Northeast, EC incidence was stable, while ACE incidence first climbed at an APC of 3.14 (95% CI, 2.0 to 4.2) from 2000 to 2008 and then subsequently stabilized. Incidence of SCE declined by 2.79% annually (95% CI, −3.0 to −2.5). In the South, EC and SCE incidence declined from 2000 to 2018 (EC: APC, −0.89; 95% CI, −1.1 to −0.6; SCE: APC, −3.20; 95% CI, −3.7 to −2.7), while ACE incidence climbed by 2.10% annually (95% CI, 0.8 to 3.4) from 2000 to 2007 before decelerating to APC of 0.54 (95% CI, 0.0 to 1.1) from 2007 to 2018. Lastly, in the Midwest, EC incidence did not change significantly, SCE incidence declined by −4.20% annually (95% CI, −5.9 to −2.5) from 2000 to 2011 and stabilized thereafter, and ACE incidence rose from 2000 to 2006 (APC, 4.2; 95% CI, 2.0 to 6.4) and from 2006 to 2018 (APC, 0.78; 95% CI, 0.1 to 1.4) (Figure 5).

## Discussion

In this retrospective population-based cross-sectional study, we examined temporal trends in incidence rates of EC and its 2 most common histologic subtypes, ACE and SCE, from 1975 through 2018 using the SEER 9 and SEER 21 registries. Based on the SEER 9, EC incidence increased from 1975 to 2004 at an APC of 0.53 (95% CI, 0.4 to 0.7) and then declined significantly by 1.0% annually (95% CI, −1.3 to −0.7) until 2018. In SEER 21, we found that despite age, sex, racial and ethnic, and geographic variation, the overall incidence of EC modestly declined since 2004, with an APC of −1.25 (95% CI, −1.5 to −1.0). Specifically, SEER 9 suggested that SCE declined from 1986 to 2011 (APC, −3.28; 95% CI, −3.5 to −3.0) and then stabilized after 2011. However, in SEER 21, from 2000 to 2018, SCE incidence significantly continued to decline (APC of −2.80, 95% CI, −3.0 to −2.6). Conversely, ACE incidence increased from 2000 to 2006 (APC of 2.51, 95% CI, 1.0 to 4.0) but then stabilized.

In this study, similar to other studies, we observed a sharp increase in the incidence rate of ACE from 1975 to the early 2000s^6,7^ prior to plateauing. The surge in ACE has been attributed to the marked rise in obesity and metabolic syndrome, risk factors that are more associated with ACE than SCE.^13,14^ However, while obesity rates in the US continue to increase,^15^ ACE incidence has plateaued during the last decade. The factors associated with this stable trend of ACE are not well understood. We speculate that a possible explanation for this pattern could be a counteracting association of the declining smoking rates^16^ with the increasing obesity rates. However, tobacco use is a risk factor more closely associated with SCE than ACE and may have a limited association with ACE incidence.^17^ The role of proton pump inhibitors (PPIs) in ACE incidence remains controversial. While one may speculate that the increased use of PPIs^18^ may be associated with reduced transition of Barrett esophagus to ACE, studies show that ACE incidence continued to increase^6,7^ following the US Food and Drug Administration approval of PPIs in 1989.^19^ Some studies have shown that long-term use of PPIs is associated with increased risk of ACE.^20,21,22,23,24^ One possible explanation is that patients have fewer symptoms and are less aware of their carcinogenic bile reflux with PPI use. More studies are needed to understand their role in ACE incidence.

The rates of upper endoscopies have increased over the years^25^ in the US, which could potentially be associated with an increase in the identification of premalignant lesions. Surprisingly, we did not observe an increase in the incidence of localized disease in EC, which would be expected if more premalignant lesions were identified through upper endoscopies. Instead, the incidence of localized disease in EC decreased or stabilized since the early 2000s. Further, we did not find evidence of stage migration. In the subgroup analysis by stage, we found that the incidence of regional and distant EC generally decreased or stabilized since the early 2000s, a change from the previously observed upward trend in SEER-based analysis from 1975 to 2006.^7^

In a subgroup analysis by race and ethnicity, age group, and sex, we found that non-Hispanic White male individuals overall exhibited the fastest rate of increase in EC and ACE at any point compared with other racial and ethnic groups. This finding has been reported in other studies that found that non-Hispanic White male individuals showed higher rates of ACE than other racial and ethnic groups.^6^ Studies to understand the reason for the higher incidence in this group are needed, although the higher incidence of GERD and Barrett esophagus, which predisposes to ACE, in this population likely plays a role.

Treatment options with the addition of immunotherapy are improving overall survival for EC,^3^ but the prognosis for EC remains poor.^2^ Therefore, we hope these findings will motivate public health interventions to reduce exposure to modifiable risk factors for EC. Interventions targeting obesity, metabolic syndrome, and smoking may decrease the incidence of EC.

### Strengths and Limitations

This study had several strengths and limitations. First, to our knowledge, this study is the most updated, nationwide, population-based analysis of the incidence of EC, ACE, and SCE in the US. Second, the study contains the largest cohort and reports on 44 years of data, the longest period studied. Third, 1 limitation is that we did not include the Asian and Other Pacific Islander individuals in the subgroup analysis because of the small sample size in the registries, so we were unable to explore incidence trends among this racial group. Fourth, we selected the 3 age group categories (<65 years, 65-75 years, and >75 years) to divide the cohort evenly for the subgroup analysis. Therefore, we may have obtained different subgroup incidence trends if we had divided the age groups differently. Also, given the limited number of patients younger than 50 years, we were unable to create a subgroup to analyze young-onset EC, as it would have resulted in insufficient statistical power to have drawn conclusive results. Fifth, the SEER 9 and SEER 21 registries do not have data from all the states; however, the registries cover 9.4% and 36.7% of the US population, respectively, and provide one of the best representative US cancer data registries. Sixth, the SEER database did not include information on GERD, Barret esophagus, or obesity. Therefore, we were unable to describe the prevalence of these factors during different periods. Finally, the SEER database has limited individual level data and does not include detailed clinical characteristics (ie, performance status) or specific treatment information (ie, chemotherapy regimens) to conduct individualized or causal inference analyses.

## Conclusions

In this population-based cross-sectional analysis from 1975 to 2018, we found that the incidence of EC and SCE continue to decrease while the incidence of ACE stabilized. Further studies are needed to identify the factors contributing to these patterns in incidence, particularly why the ACE incidence has plateaued for more than a decade and has not continued to decrease like SCE.

## Notes

Supplement 1. **eFigure.** Heatmap of Joinpoint Analysis of Esophageal Cancer (EC), Adenocarcinoma of Esophagus (ACE), and Squamous Carcinoma of Esophagus (SCE) Incidence by Age Group, Sex, and Race (2000-2018; SEER 21)

Supplement 2.
Data sharing statement
