# Supplementary material for: Trends in Epidemiology of Esophageal Cancer in the US, 1975-2018
Source: JAMA Netw Open. 2023 Aug 22;6(8):e2329497. doi: 10.1001/jamanetworkopen.2023.29497 (PMC10445206; doi:10.1001/jamanetworkopen.2023.29497)
Supplement: Supplement 1. — eFigure. Heatmap of Joinpoint Analysis of Esophageal Cancer (EC), Adenocarcinoma of Esophagus (ACE), and Squamous Carcinoma of Esophagus (SCE) Incidence by Age Group, Sex, and Race (2000-2018; SEER 21) [file jamanetwopen-e2329497-s001.pdf]

# Supplemental Online Content

Rodriguez GM, DePuy D, Aljehani M, et al. Trends in epidemiology of esophageal cancer in the US, 1975-2018. *JAMA Netw Open*. 2023;6(8):e2329497. doi:10.1001/jamanetworkopen.2023.29497

**eFigure.** Heatmap of Joinpoint Analysis of Esophageal Cancer (EC), Adenocarcinoma of Esophagus (ACE), and Squamous Carcinoma of Esophagus (SCE) Incidence by Age Group, Sex, and Race (2000-2018; SEER 21)

This supplemental material has been provided by the authors to give readers additional information about their work.

© 2023 Rodriguez GM et al. *JAMA Network Open.*

eFigure: Heatmap of Joinpoint Analysis of Esophageal Cancer (EC), Adenocarcinoma of Esophagus (ACE), and Squamous Carcinoma of Esophagus (SCE) Incidence by Age Group, Sex, and Race (2000-2018; SEER 21), *: $p<0.05$

© 2023 Rodriguez GM et al. *JAMA Network Open.*

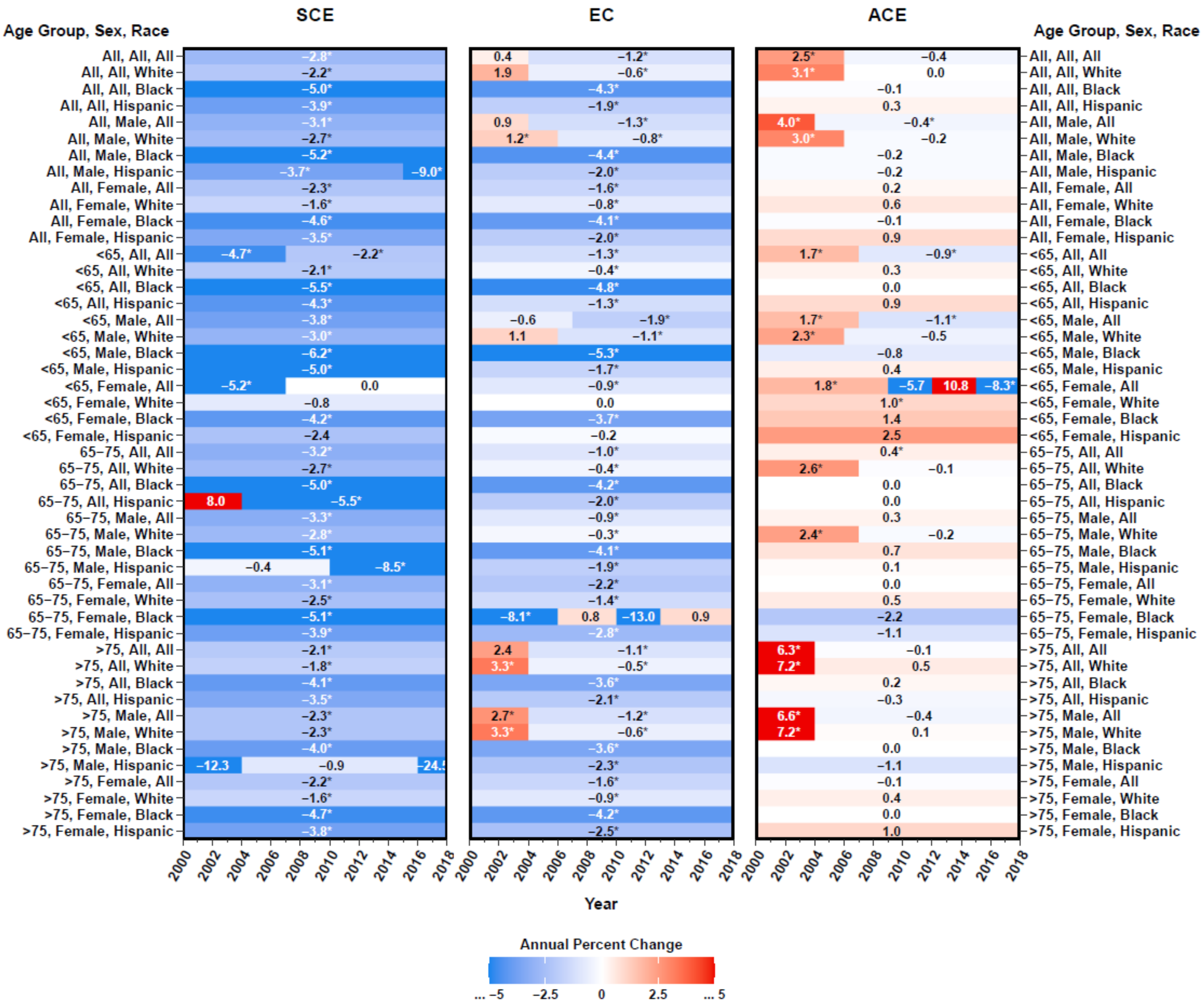


© 2023 Rodriguez GM et al. *JAMA Network Open.*
