# Supplementary material for: Trends in Epidemiology of Esophageal Cancer in the US, 1975-2018
Source: JAMA Netw Open. 2023 Aug 22;6(8):e2329497. doi: 10.1001/jamanetworkopen.2023.29497 (PMC10445206; doi:10.1001/jamanetworkopen.2023.29497)
Supplement: Supplement 2. — Data sharing statement [file jamanetwopen-e2329497-s002.pdf]

Data Sharing Statement

Rodriguez. Trends in Epidemiology of Esophageal Cancer in the US, 1975-2018. *JAMA Netw Open*. Published August 17, 2023. doi:10.1001/jamanetworkopen.2023.29497

Data
Data available: No

Additional Information
Explanation for why data not available: In this study, we used the SEER dataset which is publicly available. We did not collect any data for this study. The data we used for this study is available in the following link: https://seer.cancer.gov/data/access.html
